# Effectiveness of home-based directly observed treatment for tuberculosis in Kweneng West subdistrict, Botswana

**DOI:** 10.4102/phcfm.v2i1.168

**Published:** 2010-10-22

**Authors:** Diulu Kabongo, Bob Mash

**Affiliations:** 1 Department of Family Medicine and Primary Care, Stellenbosch University, South Africa

**Keywords:** adherence, Botswana, directly observed treatment, district health systems, home-based care, Tuberculosis

## Abstract

**Background:**

Tuberculosis (TB) and HIV are major public health problems in Botswana. In the face of growing TB notification rates, a low cure rate, human resource constraints and poor accessibility to health facilities, Botswana Ministry of Health decided to offer home-based directly observed treatment (DOT) using community volunteers.

**Objectives:**

The aim of this study was to assess the outcomes of home-based directly observed treatment (HB-DOT) versus facility-based, directly observed treatment (FB-DOT) in the Kweneng West subdistrict in Botswana and to explore the acceptability of HB-DOT among TB patients, community volunteers and health workers.

**Method:**

A quantitative, observational study using routinely collected TB data from 405 TB patients was conducted and combined with 20 qualitative in-depth interviews.

**Results:**

The overall cure rate for smear-positive pulmonary TB patients was 78.5%. Treatment outcomes were not statistically different between FB-DOT and HB-DOT. Contact tracing was significantly better in FB-DOT patients. Interviews revealed advantages and disadvantages for both FB and HB options and that flexibility in the choice or mix of options was important. A number of suggestions were made by the interviewees to improve the HB-DOT programme.

**Conclusion:**

HB-DOT is at least as good as FB-DOT in terms of the treatment outcomes, but attention must be given to contact tracing. HB-DOT offers some patients the flexibility they need to adhere to TB treatment and community volunteers may be strengthened by ongoing training and support from health workers, financial incentives and provision of basic equipment.

## INTRODUCTION

Botswana has one of the highest Tuberculosis (TB) notification rates in the world (509 cases per 100 000), with recent increases blamed mainly on the HIV epidemic. Multidrug-resistant TB (MDR TB) cases are also on the rise and 60% – 86% of TB patients are co-infected by HIV.^1, 2^ In 1975, in an attempt to halt the rapid spread of TB in Botswana, the Ministry of Health established the Botswana National Tuberculosis Control Programme (BNTP). Following this, short-course chemotherapy was introduced in 1986 and the Directly Observed Treatment Short-course (DOTS) strategy was adopted in 1993.^1^

Despite the introduction of DOTS, the TB programme continued to struggle with low cure rates, human resource constraints and difficult access to health facilities. The Botswana Ministry of Health therefore decided to offer home-based care using volunteers or family members.^2, 3, 4^ This programme allows for the dispensing of medications to TB patients at their respective homes under the daily supervision of a community volunteer. After consultation with community representatives in their respective catchment areas, health facilities select local volunteers to be trained in TB treatment supervision. At the onset of TB treatment, patients are introduced to both home-based directly observed treatment (HB-DOT) and facility-based directly observed treatment (FB-DOT) and are given the choice to continue with whichever of these methods they prefer.^2^

In Africa, there is a tradition of communities, and especially women, taking care of family members.^2^ The World Health Organization (WHO), through its Stop TB strategy, recognises community participation as one of the principles of the Primary Health Care approach.^2, 4^ The model has been successfully introduced for home-based care with HIV patients and, in the case of TB, possible contributions are^2, 5^:

- supporting patients throughout the treatment
- patient, family and community education on TB
- case finding and case detection
- recognition of adverse effects and follow up of defaulters.

Countries that have well-functioning TB programmes can also benefit from community-based approaches, as these may help them maintain their standards in the face of growing human resource deficiencies.^3^ Community involvement in dispensing anti-tuberculosis treatment has been tried in urban and rural African and Asian settings.^3, 6, 7, 8, 9, 10, 11, 12, 13^

Studies have used different community members, such as family members, volunteers, previous TB patients or established community home-based care programmes with encouraging results.^7, 8, 9, 12, 13, 14, 15^ Community TB care seems to be accepted by patients because it helps improve their understanding of TB and reduce stigma in the community.^5, 9, 16^ Finally, community TB care can help to improve the cost-effectiveness of TB treatment, although there remains an issue of incentives for volunteers to help sustain the programme.^2, 17, 18^

In Botswana, there has, to date, been little formal evaluation of the home-based programme. As such, the aim of this study was to assess the success of HB-DOT versus FB-DOT in the Kweneng West subdistrict and to explore the acceptability of HB-DOT amongst TB patients, TB treatment supervisors and health workers.

## ETHICAL CONSIDERATIONS

Ethical approval was received from the Human Research Ethics Committee, University of Stellenbosch, as well as from the Ministry of Health and Local Government in Botswana. A waiver of informed consent was obtained for use of routinely collected TB data. All interviewees signed written informed consent. There were no other potential hazards or health risks to the participants.

## METHOD

### Study setting

The Kweneng West subdistrict, a rural area with approximately 40 000 inhabitants, comprises 23 health facilities, with one primary hospital and eight main clinics that offer TB treatment. Community volunteers dispense TB medications for a given number of patients and are supervised in their respective catchment area by clinic nurses. The volunteers are given weekly TB medication and are expected to report to the clinic nurses once per week. Nurses are responsible for managing patient records, supervising medication intake and guiding the volunteers.^1^ Nurses are also supported by health education assistants, who are members of the community themselves and through whom health practitioners and facilities can sustain a link with the communities.^1^ In the TB programme, health education assistants also help to trace treatment defaulters and record all non-institutional deaths in their respective communities.^1^ Medical doctors initiate TB treatment and offer monthly follow-ups to all TB patients.^1^

TB programme data are captured at health facilities using two tools, (1) the TB register and individual TB patient cards and (2) a manual and electronic TB register maintained at district level.^1^ In particular situations, some patients may start TB treatment under FB-DOT and end up in HB-DOT or vice versa; hence, a third mixed group (MX-DOT) has been considered in this study.

### Study design

Mixed quantitative and qualitative techniques were used to evaluate the HB-DOT system. Observational data was collected from the TB registers and patient TB cards. In addition, in-depth interviews were held with TB patients, community volunteers and facility-based nurses and health education assistants.

### Study population

Only TB patients who were registered in the main clinics from June 2006 to June 2008 were included in this study. Patients with MDR or extensively drug-resistant (XDR) TB and who were transferred in or out of these clinics during this period were excluded. Using a 5% precision with 95% confidence interval, the sample size required was estimated to be at least 381 respondents.

Interviewees were chosen purposefully from the same areas as the TB patients and were actively involved in the TB programme at the time of research. Selection of TB patients aimed for a range of age groups, both genders and individuals who were employed or unemployed. As such, the following interviews were planned with the option of further interviews should the analysis suggest the need for further exploration of themes:

- five TB patients under HB-DOT
- five TB patients under FB-DOT
- five TB community volunteers
- five health workers (three nurses and two health education assistants).

### Data collection

After a short training session, research assistants collected and captured quantitative data from the TB registers and patient cards. Research assistants were community volunteers who were already acquainted with the TB programme and related ethical issues.

Interviews were conducted by the principal researcher, who was fluent in English, with the help of research assistants who were also fluent in Setswana. All interviews were recorded on audiotape. Interview guides were created for each category of interviewees to explore their experience of the HB-DOT system. For TB patients the opening question was: ‘Can you please describe your experience of TB treatment and how you now feel now about receiving TB treatment in this way?’ For TB community volunteers the opening question was: ‘Can you please describe your experience of supervising medication for a TB patient?’ Similarly, for health workers, the opening question was: ‘Can you describe the impact of HB-DOT on the TB treatment programme in your facility?’ A list of further potential questions for each interview was also identified to ensure all aspects of the issue were adequately explored.

### Analysis

Quantitative data was collated in Excel and analysed by the Centre for Statistical Consultation at the University of Stellenbosch, South Africa. Categorical data was analysed in contingency tables and a maximum likelihood Chi square test. Analysis of variance (ANOVA) was used for continuous variables and *p*-values obtained from a Kruskal–Wallis test. Transcripts of the translated tapes were prepared in English and qualitative data analysis followed the steps of the framework method,^19^ which are (1) familiarisation, (2) create a thematic index, (3) indexing, (4) charting and (5) mapping and interpretation.

## RESULTS

### Quantitative results

The study included 405 TB patients whose characteristics are shown in Table 1. The mean age was 36.5 ± SD years and did not differ significantly between the categories of TB patients (*p* = 0.52).

**TABLE 1:** The characteristics of study participants in different DOT types

Variables		All	FB-DOT	HB-DOT	MX-DOT	*p*-value

		*N* = 405	*n* = 279	*n* = 95	*n* = 31	
		***n* (%)**				

Gender	Male	220 (54)	160 (57)	46 (48)	14 (45)	0.18
	Female	185 (46)	119 (43)	49 (52)	17 (55)	
	Positive	191 (47)	131 (47)	43 (45)	17 (55)	
HIV status	Negative	171 (42)	124 (44)	35 (37)	12 (39)	0.13
	Unknown	43 (11)	24 (9)	17 (18)	2 (6)	
	Yes	75 (39)	48 (37)	19 (44)	8 (47)	
On HAART*	No	68 (36)	52 (40)	11 (26)	5 (29)	0.26
	Unknown	48 (25)	31 (23)	13 (30)	4 (24)	
Location of TB	Pulmonary	322 (80)	216 (77)	82 (86)	24 (77)	0.15
	Extra- pulmonary	83 (20)	63 (23)	13 (14)	7 (23)	
	Positive	121 (38)	92 (43)	24 (29)	5 (21)	
Initial AAFB†	Negative	94 (29)	63 (29)	21 (26)	10 (42)	0.14
	Not done	107 (33)	61 (28)	37 (45)	9 (37)	
ATTcategory	New	350 (86)	241 (86)	95 (100)	14 (45)	<0.01
	Retreatment	55 (14)	38 (14)	0 (0)	17 (55)	

DOT, directly observed treatment; ATT, anti-tuberculosis treatment; FB-DOT, facility-based, directly observed treatment; HB-DOT, home-based directly observed treatment; MX-DOT, mixed group.

* Highly active anti-retroviral treatment (HAART) is only given to patients who are HIV-positive.

† The acid- and alcohol-fast bacilli (AAFB) test is only conducted on patients who have pulmonary TB.

The TB programme encourages health workers to perform smear testing for the presence of acid- and alcohol-fast bacilli (AAFB) in diagnosing pulmonary TB, in order to avoid excessive reliance on chest radiography. In this study, 107 (33%) of patients did not have an AAFB recorded prior to treatment, which is presumably related to the inclusion of extra-pulmonary cases, but could also be a reflection on the adherence of the TB clinics to the BNTP. Similarly, 43 (11%) of patients did not have their HIV status determined.

Table 1 also compares the characteristics of the study participants in terms of the different types of DOT that they experienced. The late introduction of HB-DOT in some areas of the subdistrict may have contributed to the uneven enrolment of the different DOT types. The groups receiving different DOT types did not differ statistically in any of the characteristics, except in the category of TB treatment. None of the re-treatment patients were considered for exclusive HB-DOT, while 17 (31%) received MX-DOT. The main reason for this difference is the need for re-treatment patients to receive streptomycin injections during the intensive phase of treatment.

Table 2 compares the outcome of TB treatment across the three types of DOT and the efforts to trace TB contacts. Overall, the successful treatment rate (i.e. the sum of the cured and completed cases) was 83%. Excluding the re-treatment patients from the comparison of the types of DOT does not alter the overall findings regarding treatment outcomes and contact tracing.

**TABLE 2:** Comparison of overall outcome of TB treatment and contact tracing efforts among the different directly observed treatment types

Variables		All	FB-DOT	HB-DOT		*p*-value

		*N* = 405	*n* = 279	*n* = 95	*n* = 31	
		***n* (%)**				

	Cure	95 (23)	71 (25)	19 (20)	5 (16)	0.45
Treatment outcome	Complete	244 (60)	164 (59)	58 (61)	22 (71)	
	Died	52 (13)	34 (12)	15 (16)	3 (10)	
	Interrupted	9 (2)	8 (3)	1 (1)	0 (0)	
	Failure	5 (1)	2 (1)	2 (2)	1 (3)	
Contact tracing	Yes	245 (60.5)	190 (68)	48 (50.5)	7 (22.5)	< 0.01
	No	160 (39.5)	89 (32)	47 (49.5)	24 (77.5)	
						

DOT, directly observed treatment; FB-DOT, facility-based, directly observed treatment; HB-DOT, home-based directly observed treatment.

The outcome of treatment did not differ significantly between any of the three groups; however, health workers in the FB-DOT performed better in efforts to trace contacts of TB patients.

Table 3 shows the cure rate in terms of the total number of patients who had an initial positive AAFB (i.e. those pulmonary TB patients whose smear tested positive) as opposed to all registered patients. Again, there was no significant difference between the DOT types.

**TABLE 3:** Comparison of cure rates for smear positive pulmonary TB patients among different directly observed treatment types

	All	FB-DOT	HB-DOT	MX-DOT	*p*-value

	*n* = 121	*n* = 92	*n* = 24	*n* = 5	
Number of cured patients (cure rate)	95 (78.5%)	71 (77.2%)	19 (79.2%)	5 (100%)	0.48

DOT, directly observed treatment; FB-DOT, facility-based, directly observed treatment; HB-DOT, home-based directly observed treatment; MX-DOT, mixed group.

### Qualitative results

#### Advantages of facility-based DOT

Some patients believed that the stricter environment of the clinic was more likely to ensure their ongoing adherence. One of the interviewees expressed this belief:

*‘Because I feared getting tired of taking tablets and therefore become tempted to hide them or throw them instead of taking them. At clinics I took them in the presence of the health care worker’*.

Others felt more secure knowing that more highly trained staff were theoretically available to them at the FB-DOT service. As such, one of the interviewees who participated in FB-DOT said:

*‘I feel safe when I know the doctor is nearby and can intervene anytime if something goes wrong’*.

This was further corroborated by health care workers who felt that problems were more likely to be detected early in FB-DOT:

*‘On-going counselling, direct supervision by highly trained health workers, daily assessment of patients and therefore early detection of toxicity and side effects signs are of great importance’*.

#### Disadvantages of facility-based DOT

Patients and community volunteers recognised that FB-DOT involved longer travel time and distances, as well as longer waiting times for treatment at the clinic. Therefore, the effort and time needed to obtain treatment at clinics might adversely affect adherence, especially at weekends and during public holidays. A patient asserted:

*‘Weekly or fortnightly supply may help their experience be better. Having extra manpower over weekends at clinics may reduce the nightmare faced by a single nurse and all patients’*.

Similarly, another volunteer opined that:

*‘Patients may choose to stay home and not go late to far away health facilities’*.

Another patient commented that:

*‘Waking up early in the morning to queue at the clinics is difficult and inconveniencing, we don't get enough rest’*.

#### Advantages of home-based DOT

Obtaining treatment from a community volunteer was less stressful, as it required less effort and a more convenient and flexible time schedule could be negotiated between the patient and volunteer.

A patient under HB-DOT submitted:

*‘[HB-DOT] allows us to rest enough and does not stress us as we would have usually agreed with volunteers about a specific time to meet’*.

HB-DOT was also an advantage for those too sick to travel every day to a health facility and for those who struggled to eat before taking medication at the clinics; not being able to eat might lead to more side effects and reduce adherence. For example, a patient said:

*‘Community TB programme has come on time to help us. At times we are too sick to walk to the clinics everyday. Clinics advise us to eat before medication intake but by the time we take tablets at clinics, the food would have dissolved in the stomach. At home the volunteer deals with few people and gives time to patients to eat some porridge in the morning before medication’*.

One volunteer interviewed illustrated the point that HB-DOT allows patients to engage more fully in other aspects of their lives:

*‘[HB-DOT] is well designed to help workers or non-workers to take tablets at an agreed time with their community supervisors. Patients may then have time to take care of other businesses instead of staying at clinics the entire day’*.

Nurses also recognised these advantages and, in addition, perceived that their own workload and the need for them to conduct home visits was reduced. They also recognised that attending a health facility might place an additional financial burden on the patient:

*‘Of course HB-DOT reduces the travelling distance to clinics for patients, it reduces workload for nurses, it reduces the daily transport cost of very ill patients to/from clinics for medication or the need for home visits by nurses and the disturbance of visiting patients at home when they are still asleep’*.

HB-DOT was also an advantage on weekends when clinics may be closed.

#### Disadvantages of home-based DOT

Patients feared that being treated for TB under HB-DOT could affect their adherence to medication:

*‘We are however sometimes worried about our own safety as volunteers are perceived to be not fully knowledgeable about doses and side effects of medication for instance’*.

Health care workers concurred with these fears and the possibility that volunteers were not sufficiently knowledgeable, which led to one health care worker admitting that:

*‘[p]atients may also have little trust in volunteers’* and that

*HB-DOT unfortunately involves volunteers that are not perfect about TB management and may still have problems with some aspects of TB management. There are also concerns about the change of time of medication intake’*.

Health workers also worried that community volunteers may not always supervise strictly in the long term:

*‘Patients may relax and volunteers may be lenient to members of their community’*.

A further problem is that there is sometimes no substitute when community volunteers are not available; there is

‘[*n*]*o back up plan when volunteers have funerals or are ill’*.

Volunteers, however, suggested that health workers pay regular visits to communities where they could continue health education and reinforce what volunteers had taught:

*‘Nurses need not abandon us with the patients during the entire course of treatment. They need to meet TB patients and communities regularly to re-enforce our education. It may even increase confidence of patients in us as nurses would confirm what we usually say’*.

#### Organisational issues

Interviews with patients on FB-DOT frequently revealed that they had incomplete knowledge about the existence of HB-DOT. Sometimes they did not know that they were free to choose whether to receive medication at home or from health facilities:

Interviewer: *‘Did you know that at home you would take TB tablets in the presence of the community volunteers’?*

Patient: *‘Is it? I didn't know. I thought I would just get a supply for few days and then swallow them at my own time’*.

Problem patients on HB-DOT may be referred back to the facility when supervision is difficult. As a volunteer noted:

*‘It is discouraging to look after patients who refuse to take medicines (hide them or throw them) or patients who still take alcohol. Fortunately, difficult patients may be referred back to health facilities for follow up’*.

In addition, patients might also take their medication at the home of the community volunteer, which allows supervision to be more time-efficient for the volunteers:

*‘Patients walking to our places [volunteer's house] for medication has made supervision very easy and gives us time to finish this work and deal with our personal activities’*.

Though not specifically allowed by the national TB programme, the provision of such flexibility allows a combination of facility-, home- and even school-based supervision that can support adherence and quality of life. The advantages of this flexibility were noted by a schoolboy undergoing TB treatment:

‘I *felt that taking TB tablets was disturbing my school attendance, hence I allowed my teacher to keep my tablets and issue them to me daily. During weekends I instead take medication from the clinic. I think this arrangement is advantageous to me’*.

Nevertheless, the need for even greater flexibility was still an issue for some patients:

*‘How can I go to plough the field some 25 km away from here if everyday I have to take the TB tablets in the presence of a supervisor? What am I going to eat this year? Why don't they allow farmers to go back to their work when they feel a bit better?’*

Although relying on unpaid volunteers appears to make HB-DOT cheaper, its long-term sustainability may be threatened by volunteerism, as a volunteer points out:

*‘HB-DOT won't be sustainable as there is high turnover of volunteers due to no incentive. National TB programme should tackle this issue urgently’*.

Despite this issue, community volunteers perceive the community TB programme to be acceptable to patients:

*‘It may go a long way, if a few problems are managed, as communities have generally accepted us and take us like nurses’*.

Volunteers also appreciate the privileged position that their role in HB-DOT allows them to attain in the community, but they agree that they need to be provided with some form of identification. Indeed, identification and other clothing/equipment will ease their movement and encourage them to continue their involvement in this programme. Three different volunteers suggested:

*‘Do you know that sometimes even clinic staff fails to identify us. We need uniform and some form of sticker to write our names on to help people identify us’*.

*‘People take us as nurses and that inspires respect, why then not have identification?’*

*‘We need umbrellas and other protective cloths/boots to wear when it is raining, cold or very hot’*.

Health workers believe that the national TB programme has to take some bold decisions before HB-DOT collapses. Among their recommendations for improvement were needs for:

- financial incentives for volunteers
- training of additional volunteers to cover more areas
- organisation of regular refresher workshops for volunteers
- regular supportive visits from the district TB coordinator, local government and Ministry of Health officials to encourage them and make them feel appreciated
- constant availability of data capturing/monitoring tools
- reliable transportation to allow daily home visits of TB patients by nurses (especially when injections are required).

## CONCLUSION

Overall the choice of DOT appeared to be based on the need for injections, patient preference and availability of different DOT options (Table 4).

**TABLE 4:** Summary of the reasons for choosing different directly observed treatment types

Reasons to choose facility-based DOT	Reasons to choose home-based DOT
1. Need to obtain injections	1. Live very far from clinic
2. Feel more secure being treated by a nurse than a volunteer	2. Too weak to travel
3. Live close to a clinic	3. Can't afford to travel to clinic
4. Not too weak to travel	4. Easier to eat at home before taking treatment
	5. Flexible for workers/students

DOT, directly observed treatment.

## DISCUSSION

### Main findings of the study

There was no difference in TB treatment outcomes between facility-based, home-based and mixed types of DOT. It would appear, therefore, that HB-DOT is as good as FB-DOT. It is likely that providing greater choice and matching the treatment plan more closely with patient preferences will lead to greater satisfaction and adherence to treatment.

The overall cure rate for smear-positive pulmonary TB was 78.5%, which is below the WHO target as well as the Botswana national TB programme target of 85%. The successful treatment rate was 83%, which is close to the WHO target of 85% and above the Botswana target of 75%.

Facility-based health workers were found to be more proactive in contact tracing than the community volunteers. Given the volunteers’ greater engagement and involvement in the community, this is an unexpected finding. Further evaluation is required to explore whether this poor performance is a reflection of a lack of trust in the relationship between patients and community volunteers, or a lack of knowledge among community volunteers about contact tracing. Patients on MX-DOT showed even worse contact tracing, perhaps because no single caretaker took the responsibility of screening close contacts with TB patients.

### Relation of findings to literature

The findings of this study concur with those obtained for community-based DOT from clinical trials and observational studies conducted in different settings^3, 6, 8, 10, 20^ and support the implementation of this type of DOT as a complementary intervention to traditional FB-DOT.

Other studies demonstrated that outcomes from HB-DOT can be better than FB-DOT.^7, 9^ These studies in Zambia and Uganda showed that, in TB programmes where FB-DOT was usually only strictly implemented during the initial phase of treatment, the introduction of HB-DOT significantly improved outcomes. Other programmes have attempted to use family members instead of community volunteers for supervision. In a developed country, the use of family members showed worse adherence to anti-tuberculosis treatment (ATT) in the HB-DOT group,^14^ but in a developing country, better adherence to ATT suggests this may be an option to explore in this context.^15^

The qualitative findings are similar to those reported from Tanzania,^16^ which emphasised the benefit of HB-DOT in relieving overcrowding in clinics, as well as in providing a cost-effective, flexible and time-conscious method of treatment. Another study in Uganda also supports the link between patients’ own choice of DOT type and their commitment to completing ATT.^9^

### Strengths and weaknesses of study

A particular strength of this study is the combination of quantitative methods with exploratory qualitative interviews, which captured a more complete picture of the process and the outcome of care. This study also reports results from the actual health care setting, rather than from the more ideal conditions created by a randomised clinical trial. The MX-DOT group was not expected and its analysis was therefore affected by the small sample size.

However, a limitation of this study is the potential effect of measured and unmeasured confounding factors on outcomes between the different groups. In this study, only confounding factors registered in the TB registers were considered. Other confounding factors that may have been relevant but were not available were CD4 count, WHO clinical HIV staging and other co-morbidities.

It is important to recall that interviews are, by nature, subjective and contain the potential for misinterpretation of meaning through the process of translation and analysis.

### Need for future research

Further evaluation of HB-DOT could investigate the surprising difference in contact tracing efforts across the different DOT types, as well as the benefits of more flexibility and choice for patients. Further investigations into these elements could result in a re-evaluation of the community TB programme after addressing some of the issues raised by this study. Although the HB-DOT programme might be improved by considering the suggestions made in the interviews, the following points do need to be taken into account:

- Health care workers need to speak to TB patients on a regular basis to reinforce on-going counselling by community volunteers.
- A focus on continuing professional development needs to be sustained and a education of volunteers must be priority to ensure their skills and knowledge are competent and up-to-date.
- Additional education and support for contact tracing by community volunteers needs to be provided.
- The community volunteers should be provided with a uniform and some form of identification to reinforce their position.
- Financial incentives for community volunteers need to be considered.

All of these suggestions would increase the cost of home-based DOT and so future studies should also analyse the programme from a cost-effectiveness perspective.

## CONCLUSION

HB-DOT is at least as good as FB-DOT in terms of the treatment outcomes, but attention must be given to contact tracing. HB-DOT offers some patients the flexibility they need to adhere to TB treatment and community volunteers might be strengthened by ongoing training and support from health workers, financial incentives and provision of basic equipment.
